# Relational solidarity and COVID-19: an ethical approach to disrupt the global health disparity pathway

**DOI:** 10.1080/11287462.2021.1898090

**Published:** 2021-03-15

**Authors:** Anita Ho, Iulia Dascalu

**Affiliations:** a Centre for Applied Ethics, University of British Columbia, Vancouver, BC, Canada; b Centre for Health Evaluation and Outcome Sciences (CHÉOS), Vancouver, BC, Canada; c Bioethics Program, University of California, San Francisco, USA; d Faculty of Medicine, University of British Columbia, Vancouver, BC, Canada

**Keywords:** COVID-19, pandemic, disparity, solidarity, equity, social determinants of health

## Abstract

While the effects of COVID-19 are being felt globally, the pandemic disproportionately affects lower- and middle-income countries (LMICs) by exacerbating existing global health disparities. In this article, we illustrate how intersecting upstream social determinants of global health form a disparity pathway that compromises LMICs’ ability to respond to the pandemic. We consider pre-existing disease burden and baseline susceptibility, limited disease prevention resources, and unequal access to basic and specialized health care, essential drugs, and clinical trials. Recognizing that ongoing and underlying disparity issues will require long-term correction efforts, this pathway approach is nonetheless helpful to inform ethical responses to this global pandemic. It can facilitate international cooperation during the pandemic to reduce the disparate burdens among different regions without imposing significant burden on any particular contributor. The pathway approach allows international stakeholders in various social positions to respond to different components of the pathway based on their respective strengths and resources to help break the cycle of global health inequity. Guided by the ethical principles of relational and pragmatic solidarity, we argue for a coordinated global division of labor such that different stakeholders can collaborate to foster equitable healthcare access during this pandemic.

## Introduction

Since the first known death from COVID-19 in January 2020, the disease caused by the virus SARS-CoV-2, at least 102 million people worldwide have been infected, with over 2.2 million known deaths (Worldometer, 2021). While high-income countries (HICs) and low- and middle-income countries (LMICs) alike continue to fight the ongoing pandemic, global disparity across many domains of public health and healthcare systems are exacerbating disproportionate burden of COVID-19 in LMICs (Gostin & Friedman, 2020; Ho & Dascalu, 2020). Not only do LMICs face higher baseline disease burden due to existing endemic diseases and inadequate access to basic needs such as nutrition and sanitation; infection prevention measures are also more challenging to implement as a result of crowded living conditions and inadequate access to resources such as personal protective equipment (PPE) and testing. Furthermore, healthcare access is often limited in LMICs, which may also lack infrastructure, resources, and geopolitical power to counter international patent restrictions or competitive bidding to develop or procure treatments and vaccines. These structural barriers can exacerbate worse outcomes for people in these regions and prolong the pandemic for all in our globalized and interdependent world.

In this article, we illustrate how intersecting upstream social determinants of global health form a structural disparity pathway that compromises LMICs’ ability to respond to the pandemic. In particular, we consider pre-existing disease burden and baseline susceptibility, limited disease prevention resources, and unequal access to basic and specialized health care, essential drugs, and clinical trials. Recognizing that ongoing and underlying disparity issues will require long-term correction efforts, this pathway approach is nonetheless helpful to inform ethical responses to this global pandemic. It can facilitate different forms of international collaboration during the COVID-19 pandemic to reduce the disparate burdens among various regions without imposing significant burden on any particular contributor. The pathway approach allows international stakeholders in various social positions – including governments, civil society organizations, pharmaceutical/device companies, relevant industries, and academic/research institutions – to respond to different components of the pathway based on their respective strengths and resources to help identify and fulfill essential needs, thereby breaking the cycle of global health inequity (Adler & Newman, 2002; Kones et al., 2019). Guided by the ethical principles of relational solidarity and pragmatic solidarity, we argue for a coordinated global division of labor. Such an approach advocates for different stakeholders to collaborate to foster equitable healthcare access during this pandemic and promote long-term structural changes that can remedy the inequities that underlie and perpetuate health disparities.

## Existing global disparities in health & baseline susceptibility

In recent years, as a result of the rise of the social determinants of health (SDH) discourse, there has been increasing attention to the need of addressing social production and reproduction of health, both domestically and globally (Krumeich & Meershoek, 2014). While illnesses such as COVID-19 have biological causes (e.g. SARS-CoV-2), the SDH discourse reminds us that it is an extensive and diverse range of social variables that explain how the illness is distributed unevenly within a domestic population or globally. Such discourse highlights the bioethical concerns of ongoing global disparity, since SDHs such as country of origin, socio-economic status, gender, age, ethnicity, living and working environment, as well as domestic public and social policies are morally arbitrary. People do not deserve to be born into a high- or low-income family or region, or as a member of a particular ethnic group, such that they are not morally entitled to more or less of the benefits from various domestic or global social institutions based on these morally irrelevant factors (Rawls, 1999). Even though people may have *some* control over their living and working environment, the degree of choice is often limited (Whitehead, 1992), particularly in LMICs that may have finite educational opportunities and/or high unemployment rates. People also have minimal control over the politics of the region or the world at an individual level, even when government structures, geopolitical factors, international trade relations and policies, and patent regulations can all affect global distribution of disease burdens (Labonté et al., 2011). As some have highlighted, unfair distribution of power, money, and resources as well as the conditions of everyday life in some regions have led to marked health inequities around the world (Friel & Marmot, 2011).

Despite significant improvements in health generally in the last few decades, disparate health outcomes between HICs and LMICs linger. In the context of COVID-19, evidence shows that the aforementioned morally arbitrary factors have contributed to some people or populations having a higher risk of being exposed to SARS-CoV-2 or becoming seriously ill when infected. Within many countries, racialized and marginalized populations are suffering from disproportionate number of cases, hospitalization, and mortality from COVID-19 (Baqui et al., 2020; Kirby, 2020; Moore et al., 2020). In the broader global context, existing disease burden in LMICs predisposes already vulnerable populations to increased COVID-19 morbidity and mortality. Comorbid conditions that correlate with increased disease severity in patients with COVID-19 include hypertension, diabetes, COPD, cardiovascular disease, and cerebrovascular disease (Wang et al., 2020; Yang et al., 2020). Two-thirds of the 1.1 billion people worldwide with hypertension live in LMICs (World Health Organization, 2019), and diabetes rates have been rising faster in LMICs than in HICs (World Health Organization, 2020c). Ongoing epidemics of HIV, tuberculosis, and malaria in LMICs, together with the new COVID-19 epidemic, will likely cause increased morbidity and mortality (Thienemann et al., 2020), as COVID-19 is diverting human and financial resources away from existing epidemics (Hogan et al., 2020; Pai, 2020). The WHO estimates that deaths from malaria in Sub-Saharan Africa could double this year due to COVID-19 related disruptions in prevention and treatment (World Health Organization, 2020b). Similar concerns abound for increasing childhood malnutrition and reduction in essential maternal and child health interventions (Headey et al., 2020; Roberton et al., 2020). Furthermore, according to GAVI, the Vaccine Alliance, more than 13.5 million people in 13 of the lowest-income countries may not receive vaccines against diseases like polio and measles due to disrupted immunization programs – a particular concern given ongoing measles outbreaks in several countries, including the Democratic Republic of the Congo and the Central African Republic (Gavi, 2020). Without adequate action, the profound impact of COVID on early life nutrition and vaccination could have intergenerational consequences for child growth and development as well as life-long impacts on education, chronic, disease risks, and overall human capital formation, thereby perpetuating global disparity (Headey et al., 2020).

## Lack of prevention and mitigation resources

Intersecting with baseline vulnerability, a lack of resources to prevent and limit disease spread, including PPE, physical distancing, handwashing, testing, and access to effective and affordable vaccines, is amplifying the burden of addressing the immediate social determinants of COVID-19 in LMICs (McMahon et al., 2020). For example, chronic poverty affects nearly 70% of the population in Haiti, which declared its first two cases of COVID-19 on March 19, 2020 (Cénat, 2020). As of September 12, 2020, Haiti’s confirmed case number is 8,478, although the low availability of tests (2.2 tests per 1000 people) likely means that many infected individuals have not been counted (Worldometer, 2020). The Pan American Health Organization has warned of an impending humanitarian crisis in Haiti, a country that has one of the highest mortality rates from natural disasters in the world due to some of the aforementioned social determinants of global health (Beaubien, 2020; Rouzier et al., 2020). As is the case in many LMICs, most Haitians lack access to potable water and sanitation, posing a significant barrier to limiting the spread of infection through handwashing. While residents in HICs with spacious housing and the ability to work from home may be able to achieve safe distancing, implementing physical distancing is nearly impossible in Haiti due to crowded living conditions (Beaubien, 2020). As almost half of the country’s working population is employed in the agricultural sector, self-isolation or work-from-home is not feasible for most Haitians (Moloney, 2020; The World Bank, 2020a). Similar concerns also face other LMICs, especially in impoverished areas where people share latrines and wells, and go to markets regularly to purchase food that cannot be stored at home, further risking exposure and propagating outbreaks (Maxmen, 2020).

Testing is another important mitigation strategy by identifying and subsequently isolating infected individuals and their close contacts. Limited availability of tests is a major issue in both HICs and LMICs, but there are vast inequities between regions and countries. While the United States conducted around 118 tests per 1,000 people as of July 10, India and Kenya respectively reported testing rates of 8 tests and 4 tests per 1,000 people (Global Change Data Lab, 2020). Low test rates may result in undercounting infected individuals, thereby stalling contact tracing, isolation, and other mitigation efforts. They can also prevent health systems from using accurate data to prepare for and build capacity for surging needs accordingly.

Worldwide PPE shortages, due partly to panic buying and irrational use of N95 respirators in HICs (Hopman et al., 2020), are also disproportionately affecting LMICs, many of which are relying on donations from organizations such as UNICEF and GAVI for both PPE and diagnostic tests (UNICEF, 2020). The lack of supplies, particularly for healthcare providers, can threaten the safety of the overwhelmed health workforce and exacerbate the risk of hospital-acquired infections that can further compromise the health of these nations.

## Disparity in health care access

The disparity pathway continues after people in LMICs become infected. Access to healthcare services was already limited in many LMICs prior to the pandemic. There are only 0.2 physicians per 1,000 people in Sub-Saharan Africa, compared to somewhere between 2.6 (Canada) to 4.2 (Germany) physicians for every 1,000 people in HICs (The World Bank, 2020b). As COVID-19 cases increase, the number of individuals requiring treatment greatly exceed system capacity. Haiti, with a population of 11 million, has only 0.7 hospital beds per 1000 inhabitants, and fewer than 30 ICU beds in the entire country (Cénat, 2020). It is estimated that there are only 9800 ICU beds available across Africa, compared to the estimated need of at least 121,000 critical care beds when the pandemic reaches its peak, amounting to one ICU bed and one ventilator per 100,000 people and perpetuating ongoing disparity in that country (Houreld et al., 2020). This is in contrast to the United States (U.S.), where there are 29.4 intensive care beds per 100,000 people (Society of Critical Care Medicine, 2020). Ventilators are also scarce in LMICs: Eritrea has no ventilators (Houreld et al., 2020), and South Sudan has just 4 ventilators and 24 ICU beds for its 12 million people (Woodyatt, 2020). In countries such as South Africa where ventilators are more available, many are in private hospitals, rendering them inaccessible for the majority of the population (Houreld et al., 2020). South Africa registered 1 million cases before the end of 2020, and the new and more contagious variant further heightens concerns that the country’s health system may collapse (Lloyd, 2021). Furthermore, ventilators rely on medical grade oxygen, electricity, and trained staff to function effectively, all of which are in limited supply. As Africa’s reported cases tripled since May 2020 (Africa CDC, 2020), including more than a two-fold increase among healthcare workers, the continent is witnessing a catastrophic shortage of medical professionals as the need for care soars (Massinga Loembé et al., 2020).

## Unequal access to drugs & clinical trials

Major efforts are being made by the global scientific community to repurpose existing medications used for other conditions to treat coronavirus “off-label,” as well as to find effective new treatments and vaccines against COVID-19. Given that many of the medications being used in COVID-19 clinical trials are already approved for other uses, ethical procurement and allocation of these drugs at the global level is of utmost importance, as repurposing medications primarily used for diseases which disproportionately affect LMICs may reinforce structural inequality and exacerbate health disparity. The initial hoarding of hydroxychloroquine by some HICs despite the lack of robust evidence to support its use for COVID-19 exacerbated fears of a drug supply shortage that would threaten the life and well-being of many patients. These medications have been the low-cost treatments for patients living with systemic lupus erythematosus and various rheumatologic diseases (Jakhar & Kaur, 2020), and the main drug for malaria patients in parts of Central America, Haiti, the Dominican Republic, and the Middle East (Center for Disease Control, 2019). While various countries subsequently revoked authorization for hydroxychloroquine for COVID-19 treatment due to lack of efficacy data (U.S Food and Drug Administration, 2020), hoarding of other drugs, most notably by the U.S., continues. Gilead donated its entire initial supply of remdesivir (140,000 treatment courses) to the U.S. government (Sternlicht, 2020), which reportedly bought 500,000 doses subsequently – almost all of the company’s production for the next three months (Boseley, 2020). The cost of over USD$3000 for a five-day course would also be prohibitive for many health systems and patients in LMICs. The U.S. also invoked the Defense Production Act to block some American-made medical supplies from being sent abroad, even as it threatened to retaliate against India earlier in the pandemic for temporarily banning hydroxychloroquine export (BBC, 2020).

Disparity also exists in clinical trial access across the globe. In March, the World Health Organization launched the Solidarity Trial, a global randomized control “megatrial” of the most promising treatments. As of July 1st, nearly 5500 patients have been recruited in 21 different countries as part of the Solidarity Trial, and over 100 countries have expressed interest in participating (World Health Organization, 2020a). However, as is often the case in international drug trials, African countries are underrepresented, and delays in securing supplies and expertise pose a barrier to conducting their own trials (Roussi & Maxmen, 2020).

This unequal access to clinical trials is concerning from both clinical and distributive justice perspectives, reflecting and simultaneously perpetuating structural global inequality, since the megatrial drugs may be difficult to obtain and administer in LMICs (Roussi & Maxmen, 2020). For example, remdesivir is administered through intravenous infusion, which may pose a challenge to conducting drug trials in LMICs with limited hospitals, medical supplies, and trained personnel. While the manufacturer announced plans to test an inhaled version of the drug, no information on clinical trials has been released as the company awaits regulatory approval. Since storage modality (e.g. refrigeration) and methods of administration (e.g. single versus multiple doses) may affect the feasibility of using some vaccines in settings that have limited electricity, clean water, or space and staff capacity, clinical trials in different regions are essential to ensure that there would be appropriate vaccines and treatments developed for diverse areas with different environmental and socio-economic realities.

Unfortunately, despite the efforts of researchers worldwide to form a global coalition to accelerate COVID-19 research in LMICs (COVID-19 Clinical Research Coalition, 2020), disparity in trial access persists. As of January 31, 2021, at least 4609 COVID-19 trials are registered as currently active around the world (U.S. National Library of Medicine, 2021). Only 237 of these trials are taking place in Africa, compared to 1705 trials in Europe. While the higher number of confirmed cases and quick viral spread in some of the HICs may partly explain the difference in regional participation and recruitment, the substantially lower investment in lower-resource regions reflect ongoing barriers to advance equitable research and associated benefits in LMICs (U.S. National Library of Medicine, 2021).

Unequal access to global vaccine development heightens concerns about disparate distribution and effectiveness of any approved vaccines. LMICs may not have the infrastructure to produce or deliver enough vaccine for their entire population or modify promising vaccines when new variants are found (Zimmer et al., 2021), and many depend on collaboration with or donation by HICs. Concerns abound that HICs are prioritizing distribution of a vaccine to their own population before exporting it globally, repeating past history of vaccine nationalism. During the H1N1 epidemic, HICs either delayed or abstained from exporting vaccine to LMICs altogether (Fidler, 2010). In the current COVID-19 pandemic, the U.S., the European Union, Japan and the U.K. have reportedly agreed to purchase at least 3.7 billion doses of vaccines upon approval from Western drug makers, with options available for additional doses. As of January 31, 2021, while more than 90 million people worldwide have been vaccinated, in sub-Saharan Africa, which is the home of approximately 1 billion people, only 25 people had been given doses outside of clinical trials (Chutel & Santora, 2021). As HICs continue to enter bilateral agreements with vaccine companies, directly and indirectly excluding poorer countries from receiving supplies, the WHO warns that the global vaccine disparity could soon become a catastrophic moral failure (Schemm & Hassan, 2021). Without explicit and committed international collaboration and accountability to prevent hoarding supplies, such nationalistic behavior by HICs could further exacerbate COVID-19 disease burden and mortality in LMICs (Wan & Johnson, 2020).

## From protectionist nationalism to global solidarity

Governments have a general responsibility as per their social contract with their domestic population to create the conditions in which people can be as healthy as possible, including (but not limited to) providing the public health infrastructure to control infectious diseases (Boufford & Cassel, 2002). Facing a novel virus that brought uncertainties and widespread local transmission, nationalistic and protectionist behavior by any country, including HICs, was understandable in the early phases of the COVID-19 pandemic when public health agencies across the world were working with imperfect and evolving information regarding their ability to use existing resources to mitigate the spread of COVID-19. Shortages of resources such as medications, PPE, and testing kits were affecting HICs and LMICs alike, and each health system has the responsibility to first use their resources to curtail local transmission while trying to expand capacity.

As the pandemic of an easily-transmittable virus persists, particularly with the discovery of new and more contagious variants, the disparity pathway illustrates how intersecting global determinants of health gradually widen the gaps between HICs and LMICs in their ability to combat COVID-19 or sustain their public health and primary care infrastructure. HICs that quickly coordinated and adopted drastic and unified mitigation strategies (e.g. New Zealand) gradually flattened the curve and are finding adaptive ways to prevent or prepare for subsequent waves. The situation in LMICs is more complex and concerning. For example, Africa has been reporting a disproportionately small fraction of the world’s caseload, partly due to less international air traffic compared to HICs and the predominantly younger age of the populations (Maxmen, 2020). Moreover, testing remains limited in some areas, such that many mild and asymptomatic cases have likely been undetected, or that patients’ illnesses may have been miscategorized as other respiratory diseases. Nonetheless, these undercounted individuals can still spread the virus, and confirmed infections are on the rise (Paquette, 2020), potentially exacerbating pressure on the under-resourced health care system.

As mutual vulnerability intersects with the health disparity pathway, putting LMICs at a disadvantage in combating COVID-19, the concept of solidarity may help guide the international community in tackling the current pandemic (Tosam et al., 2018). Solidarity is a relational concept based upon common interests and mutual advantage (Baylis et al., 2008). It is also a pragmatic concept, as solidarity requires explicit actions to reduce unnecessary and disparate suffering as well as promoting well-being for all (Farmer & Gastineau, 2002). Solidarity is thus an enacted commitment to assist others in a similar plight as moral equals with unequal resources. It entails acting on behalf of others and accepting costs “to provide accessible health care, or to accept restrictions on freedoms to consume scarce resources, to benefit them” (West-Oram & Buyx, 2017). The relational understanding of solidarity acknowledges that, despite common needs and mutual vulnerability, some populations have been exposed to more structural disadvantages and associated social injustices that require correction (Baylis et al., 2008). The pragmatic requirement of solidarity demands that we synergize health and human rights by rapidly deploying tools and resources to improve the health and well-being of populations who otherwise may lack essential access, including those who are in LMICs, where the lack of health and healthcare resources have further reduced their ability to achieve economic, cultural, and social rights (Farmer & Gastineau, 2002).

Addressing the disparity pathway through the lens of global health solidarity is particularly important from an ethical perspective. This lens provides a long-term view for structural changes that can remedy the various aspects of systemic inequities that are the key drivers of health disparity. It recognizes the equal moral status of different stakeholders who occupy varying circumstances and social positions at any given point. Global health solidarity highlights that contributions to our neighbors, close and afar, are not unidirectional aid or charity that constitutes a dependency relationship. In relational solidarity, stakeholders who face different circumstances and realities are recognized as moral equals who face similar threats to varying extent as a result of both circumstances and socio-historical or structural injustices, and can collaboratively make different contributions to help promote common interests and just allocation of resources to restore our equal right to health (West-Oram & Buyx, 2017). Recognizing the bi-directional nature of solidarity, HICs can also adopt reverse innovation by learning from successes in LMICs that may help promote creative solutions for the new pandemic. Countries such as Rwanda and Uruguay have been able to control community spread even as many HICs struggle, and LMICs’ strategies in promoting trust and aligning decision makers, scientists, and national health authorities may offer valuable lessons to all (Cahan, 2020; Taylor, 2020). Nationalism and relational solidarity can thus co-exist through the relational concept and pragmatic actions of reciprocity. Governments, civil society organizations (e.g. community groups, non-government organizations, charitable and faith-based organizations, indigenous groups, professional associations, and foundations), research institutions, and relevant industries can collaborate to allocate various types of resources to areas with the greatest need to avoid exacerbating health disparity, with the understanding that this support can be reciprocated in different ways through collaboration and co-learning as the pandemic evolves.

## Strategies to disrupt the disparity pathway

A pathway approach may increase the feasibility of social and political action to promote solidarity. It can facilitate a multi-sectoral global response to curb the exacerbation of such inequity at various points along the pathway without imposing undue burden on any particular stakeholder. In the globalized world, HICs and LMICs have become increasingly interdependent for promotion of health (Labonté et al., 2009), highlighting the imperative for collective global action (Friel & Marmot, 2011). As international stakeholders with various roles and strengths are better positioned to address different aspects of disparity, the pathway approach allows the division of corrective labor and collaborative efforts to address and minimize health disparity in this pandemic.

First, in addressing baseline susceptibility and relative lack of prevention and mitigation resources in LMICs, governments, health systems, and civil society organizations can coordinate with different industries to ensure that LMICs will be able to sustain ongoing primary care and public health efforts. As we have seen, broad scale disadvantages are exacerbating LMICs’ vulnerabilities in the COVID-19 response pathway, calling for a multi-stakeholder and multi-prong approach to help address various socio-economic and other relevant factors that may affect health and health equity. As health systems in HICs focus on ventilator or ICU bed access for their populations, civil society organizations in these regions can collaborate with regional and local counterparts in LMICs for collective efforts to sustain or improve access to primary care systems to reduce the impact of chronic conditions. This may be particularly important given the link between chronic diseases and COVID-19 outcomes (Mainous et al., 2020). Efforts to sustain or fortify primary care as well as ongoing public health interventions (e.g. immunization) likely would not require substantial new expertise, but can prevent people from facing additional vulnerabilities that may exacerbate their COVID-19 risks. As some sectors may experience higher levels of layoffs during this pandemic, resources to help train otherwise unemployed people as community health workers for outreach efforts may help to increase community involvement, build trust and health literacy, and promote low-cost mitigation programs. Financial investment or donation to keep the current health workforce employed can also minimize service disruption.

Second, as prevention services may go beyond clinical care, attention to other preventive services and basic resources may be important. Health systems and civil society organizations can coordinate with different industries to provide essential supplies such as handwashing stations and other basic hygiene supplies (e.g. soap, disinfectants) to LMICs. The World Bank and NGOs such as WaterAid, for example, are working with countries and partners to promote access to reliable water supplies and hygiene facilities (The World Bank, 2020c; WaterAid, 2020). Other industries, such as logistics, construction, electricity, automobile, or petroleum, can also be part of the concerted effort to achieve more equity (Ho, 2017). They can contribute to building shelter to facilitate physical distancing, providing vehicles and fuels for efficient patient transportation, or sustaining the infrastructure for a responsive primary healthcare and social care system. Moreover, as the virus ravages different regions at various times, extra resources produced or stockpiled by HICs can be shared with countries at the highest needs at that time. HICs can donate PPE and testing kits to countries that do not have the capacity to manufacture enough of these for their own population, or support LMICs in funding low-cost initiatives that can convert existing factories to boost mask or affordable ventilators for the host countries. Enhancing the supply chain may also help healthcare providers who are equipped with these supplies to educate patients and community members on how to procure and utilize these resources appropriately to mitigate viral spread. In the spirit of reciprocity, these coordinated efforts and low-cost initiatives may facilitate regional development that can in turn provide valuable knowledge and enhance production and education capacity that can benefit all regions as they develop potentially long-term and durable response strategies.

Third, to sustain and inform regionally responsive actions, academic and research institutions can partner with governments, health systems, policy makers, community organizations, and practitioners in fulfilling pragmatic solidarity. They can form networks of research, policy, and practice to gather and analyze pertinent data to inform evolving implementation plans and ongoing operations in various regions (Friel & Marmot, 2011). For example, international contributors can collaborate with the African Taskforce for Coronavirus (AFTCOR) to develop sectoral strategies to combat the virus and to study its impact (Nkengasong & Mankoula, 2020). Such collaboration is ethically and epistemologically important as part of moral respect and reciprocity. It gives power to LMICs that have been long marginalized in the global world and acknowledges the equal contribution from different regions as we continue to learn about the evolving pandemic. Moreover, coordinated data collection from LMICs may help all regions to learn about these countries’ best practices as well as the innovation and impact of using low-cost and low-tech resources that may be applied to other settings. For example, some West and Central African countries such as Liberia, Sierra Leone, and Guinea had recent experience combatting Ebola, and other countries in the region have existing emergency health care protocols based on other viral outbreaks (Maxmen, 2020). Vietnam’s vigorous contact tracing, strict quarantine requirements, and border control have also helped the country to control the spread of COVID-19 better than higher-resource counterparts (Lowy Institute, 2021). Their success and lessons may help to inform other countries regarding various mitigation strategies. Contextual data modeling using information from both HICs and LMICs may also help to predict the evolution of the global pandemic more accurately. In addition, online resources developed by researchers from HICs may also be adapted for or shared with LMICs, or co-developed with research and community partners as feasible to maximize regional applicability. For example, Project HOPE, an international NGO that has partnered with academic experts to provide virtual COVID-19 train-the-trainers programs around the world to enhance local capacity that will go beyond this pandemic, provides a model for promoting pragmatic solidarity (Torbay, 2020).

Fourth, and related to the last strategy, in addressing disparity in healthcare access and to promote fair distribution of benefits and burdens in pandemic research, HICs and relevant research organizations can commit to forming global research coalitions with LMICs for drug and vaccine trials as well as mechanisms for needs-based allocation (World Health Organization, 2018). The WHO has emphasized the moral importance for people around the world to have access to safe, effective, and affordable medicines and vaccines (World Health Organization, 2018). In fostering greater access to and understanding of international pools of data, global shared effort that recognizes interdependence can promote robust research and mutual advances in our understanding of the virus as it spreads and mutates around the world. In the current pandemic, global public-private collaboration in vaccine development can create more opportunities to test multiple vaccine candidates to serve people in different regions (Corey et al., 2020). As no single vaccine is likely going to meet the global need, a commitment to relational and pragmatic solidarity via international collaboration can help to coordinate harmonized clinical trials for diverse vaccine candidates and promote equitable global distribution in the pandemic.

GAVI has proposed an “advance market commitment” for a portfolio of candidate vaccines, guaranteeing to purchase vaccine doses for LMICs at a lower cost, thus allowing manufacturers to scale up production with reduced financial risk in order to then sell vaccines at a higher cost to HICs (Wan & Johnson, 2020). Within this COVID-19 Vaccines Global Access Facility (COVAX) initiative that is backed by the WHO and other partners (Cumming-Bruce, 2021; World Health Organization, 2020d), both HICs and LMICs can pledge money and receive access to a wider range of vaccines than what they might otherwise be able to access, thereby ensuring more efficient and equitable global distribution (and pricing) of the vaccines when they become available (Wan & Johnson, 2020).

Fifth, international collaborations to meet global health needs, particularly public-private partnerships, establish a moral obligation of reciprocity to ensure that the benefits are fairly allocated. We argue that the establishment of COVAX and other public-private partnerships creates more than an immediate contractual obligation for pharmaceutical companies and HICs to provide the vaccines and funding according to the terms in the specific purchasing agreements. Such partnership was formed mainly as an effort to ensure adequate access to essential vaccines, thereby bestowing a broader ethical obligation for benefiting companies to adjust patent enforcement and pricing schemes accordingly beyond the pandemic, and for governments to refrain from hoarding supplies or engaging in competitive bidding that may exclude LMICs with less financial resources from acquiring needed drugs and vaccines (Mullard, 2020). Governments, state-funded institutions, and NGOs are heavily subsidizing therapeutics and vaccine research through various means (e.g. research support, direct funding, donations, tax deductions), such that the financial burden of drug or vaccine development is not borne completely by pharmaceutical companies or a single government. The reciprocity argument suggests that pharmaceutical companies, which are benefiting from such collaboration, have a moral obligation to continue to support public agencies’ humanitarian efforts in making vaccines available to people in LMICs (Ho, 2017). Vaccine manufacturers are in the unique position to help by virtue of their products; they can control the supply and pricing, both of which would have significant impact on whether LMICs may be able to obtain adequate vaccine for their populations. Some have pointed out that pharmaceutical companies are entitled to a reasonable profit and are not obligated to distribute vaccines for free or at such low prices that they may sustain meager profits or even losses (Spinello, 1992). Nonetheless, since LMICs would not have been a lucrative market due to their economic plight, pharmaceutical companies can allow generic versions of their drugs or vaccines to be manufactured in lower-cost countries (e.g. India and Brazil) that can be parallel-exported to LMICs without significantly affecting their bottom line (Boseley, 2021).

The reciprocity argument also applies to governments, which are benefitting from the international collaboration for a wider supply of vaccines. The recognition of such ethical obligation is important to promote long-term accessibility of COVID-19 vaccines, as the COVAX program does not prohibit HICs from establishing separate contracts to acquire vaccines directly (Cumming-Bruce, 2021). As some HICs may try to vaccinate their lower-risk populations (e.g. young and healthy) while LMICs still lack access for their high-risk populations, the program’s success in delivering necessary doses around the world will likely require various nations to commit to initially only procuring what is necessary to obtain enough vaccine for their most susceptible populations, so that there will be enough vaccines to be allocated to other countries, particularly LMICs, to inoculate their most vulnerable citizens and frontline health workers (Shah, 2020). Given that any new treatment or vaccine is unlikely to be 100% protective, if HICs’ nationalist strategies halt the critical international supply lines, people fortunate to have been treated or vaccinated in HICs may still be at risk of infection from unprotected populations (MacDonald, 2009).

## Conclusion

Global disparities in the COVID-19 pandemic span the entire healthcare pathway, from baseline population risk to the respective countries’ ability to prevent disease spread and treat infected individuals. The pathway approach demonstrates how such disparities are systemic, requiring long-term and structural changes to correct the unfair and unjust geopolitical and socio-economic forces that have contributed to vast inequities. When guided by the principles of relational and pragmatic solidarity, multiple stakeholders can work together in favor of the common good, not only to disrupt the continuing international spread of COVID-19, but also to prevent the exacerbation of global injustices and health disparities. The current pandemic highlights how various countries face many common health risks to varying extent, and that the global community is interdependent in tackling these common issues (Frenk et al., 2014). The ideas of relational and pragmatic solidarity remind us that no single stakeholder can address pandemic threats unilaterally. Since many determinants of health have globalized (Frenk et al., 2014), they require a collaborative effort to reinforce any nation’s actions to contain and eliminate the virus. By disrupting the disparity pathway, solidarity that facilitates protecting more fragile regions also promotes the good for all.

## References

[CIT0001] Adler, N. E., & Newman, K. (2002). Socioeconomic disparities in health: Pathways and policies. *Health Affairs*, *21*(2), 60–76. https://doi.org/10.1377/hlthaff.21.2.60 11900187

[CIT0002] Africa CDC. (2020). Coronavirus disease 2019 (COVID-19). *Africa CDC*. Retrieved July 15, 2020, from https://africacdc.org/covid-19/

[CIT0003] Baqui, P., Bica, I., Marra, V., Ercole, A., & van der Schaar, M. (2020). Ethnic and regional variations in hospital mortality from COVID-19 in Brazil: A cross-sectional observational study. *The Lancet Global Health*, *8*(8), e1018–e1026. https://doi.org/10.1016/S2214-109X(20)30285-0 32622400 PMC7332269

[CIT0004] Baylis, F., Kenny, N. P., & Sherwin, S. (2008). A relational account of public health ethics. *Public Health Ethics*, *1*(3), 196–209. https://doi.org/10.1093/phe/phn025

[CIT0005] BBC. (2020, April 7). Hydroxychloroquine: India agrees to release drug after Trump retaliation threat. *BBC News*. Retrieved April 25, 2020, from https://www.bbc.com/news/world-asia-india-52196730

[CIT0006] Beaubien, J. (2020). Pan American health organization fears “humanitarian crisis” in Haiti. *NPR*. Retrieved May 17, 2020, from https://www.npr.org/sections/goatsandsoda/2020/05/08/853052522/haitian-doctor-says-this-is-the-worst-epidemic-hes-faced

[CIT0007] Boseley, S. (2020, June 30). US secures world stock of key Covid-19 drug remdesivir. *The Guardian*. Retrieved July 15, 2020, from https://www.theguardian.com/us-news/2020/jun/30/us-buys-up-world-stock-of-key-covid-19-drug

[CIT0008] Boseley, S. (2021, January 15). Global immunisation: low-income countries rush to access Covid vaccine supply. *The Guardian*. Retrieved February 6, 2021, from https://www.theguardian.com/world/2021/jan/15/global-immunisation-low-income-countries-rush-to-access-covid-vaccine-supply

[CIT0009] Boufford, J. I., & Cassel, C. K. (2002). The governmental public health infrastructure. In *The future of the public’s health in the 21st century* (pp. 96–177). https://www.ncbi.nlm.nih.gov/books/NBK221231/

[CIT0010] Cahan, E. M. (2020, December 11). Rwanda’s secret weapon against covid-19: Trust. *The BMJ*, *371*. https://doi.org/10.1136/bmj.m4720 33310784

[CIT0011] Cénat, J. M. (2020). The vulnerability of low-and middle-income countries facing the COVID-19 pandemic: The case of Haiti. *Travel Medicine and Infectious Disease*. https://doi.org/10.1016/j.tmaid.2020.101684 PMC7175892 32334090

[CIT0012] Center for Disease Control. (2019, April). Treatment guidelines – treatment of Malaria (guidelines for clinicians). *U.S. Centers For Disease Control and Prevention*, 1–8. http://www.cdc.gov/malaria/resources/pdf/treatmenttable.pdf

[CIT0013] Chutel, L., & Santora, M. (2021, January 31). As virus variants spread, ‘no one is safe until everyone is safe’. *The New York Times*. Retrieved February 6, 2021, from https://www.nytimes.com/2021/01/31/world/africa/coronavirus-south-africa-variant.html?action=click&auth=login-google&module=Top Stories&pgtype=Homepage

[CIT0014] Corey, L., Mascola, J. R., Fauci, A. S., & Collins, F. S. (2020, May 29). A strategic approach to COVID-19 vaccine R&D: A public-private partnership and platform for harmonized clinical trials aims to accelerate licensure and distribution. *Science*, *368*(6494), 948–950. https://doi.org/10.1126/science.abc5312 32393526

[CIT0015] COVID-19 Clinical Research Coalition. (2020). Global coalition to accelerate COVID-19 clinical research in resource-limited settings. *The Lancet*, *2*(20), 19–21. https://doi.org/10.1016/s0140-6736(20)30798-4 PMC7270833 32247324

[CIT0016] Cumming-Bruce, N. (2021, February 6). Covid-19: the world has changed. So has the playbook for the Olympics. *The New York Times.* Retrieved February 6, 2021, from https://www.nytimes.com/live/2021/02/03/world/covid-19-coronavirus#a-global-program-to-supply-vaccine-to-poorer-countries-plans-to-ship-over-300-million-doses-by-june-30

[CIT0017] Farmer, P., & Gastineau, N. (2002). Rethinking health and human rights: Time for a paradigm shift. *Journal of Law, Medicine & Ethics*, *30*(4), 655–666. https://doi.org/10.1111/j.1748-720X.2002.tb00433.x 12561271

[CIT0018] Fidler, D. P. (2010). Negotiating equitable access to influenza vaccines: Global health diplomacy and the controversies surrounding avian influenza H5N1 and pandemic influenza H1N1. *PLoS Medicine*, *7*(5), 5. https://doi.org/10.1371/journal.pmed.1000247 PMC2864298 20454566

[CIT0019] Frenk, J., Gómez-Dantés, O., & Moon, S. (2014). From sovereignty to solidarity: A renewed concept of global health for an era of complex interdependence. *The Lancet*, *383*(9911), 94–97. https://doi.org/10.1016/S0140-6736(13)62561-1 24388312

[CIT0020] Friel, S., & Marmot, M. G. (2011). Action on the social determinants of health and health inequities goes global. *Annual Review of Public Health*, *32*(1), 225–236. https://doi.org/10.1146/annurev-publhealth-031210-101220 21219162

[CIT0021] Gavi, the V. A. (2020). COVID-19: massive impact on lower-income countries threatens more disease outbreaks | Gavi, the Vaccine Alliance. *Gavi, the Vaccine Alliance.* Retrieved May 18, 2020, from https://www.gavi.org/news/media-room/covid-19-massive-impact-lower-income-countries-threatens-more-disease-outbreaks

[CIT0022] Global Change Data Lab. (2020). Total COVID-19 tests per 1,000 people. *Our World in Data*. Retrieved July 15, 2020, from https://ourworldindata.org/grapher/full-list-cumulative-total-tests-per-thousand-map?tab=table

[CIT0025a] Gostin, L., & Friedman, E. A. (2020, May 1). Health inequalities. *Hastings Center Report*, *50*(4), 6–8. 10.1002/hast.1108 PMC7267527 32356918

[CIT0023] Headey, D., Heidkamp, R., Osendarp, S., Ruel, M., Scott, N., Black, R., Shekar, M., Bouis, H., Flory, A., Haddad, L., & Walker, N. (2020, August 22). Impacts of COVID-19 on childhood malnutrition and nutrition-related mortality. *The Lancet*, *396*(10250), 519–521. https://doi.org/10.1016/S0140-6736(20)31647-0 PMC7384798 32730743

[CIT0024] Ho, A. (2017). Global health disparity and pharmaceutical companies’ obligation to assist. In D. Ho (Ed.), *Philosophical issues in pharmaceutics: Development, dispensing, and use* (pp. 29–45). https://doi.org/10.1007/978-94-024-0979-6_3

[CIT0026] Hogan, A. B., Jewell, B. L., Sherrard-smith, E., Vesga, J. F., Watson, O. J., Whittaker, C., Hamlet, A., Smith, J. A., Winskill, P., Verity, R., Baguelin, M., Lees, J. A., Whittles, L. K., Ainslie, K. E. C., Bhatt, S., Boonyasiri, A., Brazeau, N. F., Cattarino, L., & Hallett, T. B. (2020). Potential impact of the COVID-19 pandemic on HIV, tuberculosis, and malaria in low-income and middle-income countries: A modelling study. *The Lancet Global Health*, 1–10. https://doi.org/10.1016/S2214-109X(20)30288-6 PMC7357988 32673577

[CIT0027] Hopman, J., Allegranzi, B., & Mehtar, S. (2020, April 28). Managing COVID-19 in low- and middle-income countries. *JAMA*, *323*(16), 1549–1550. https://doi.org/10.1001/jama.2020.4169 32176764

[CIT0028] Houreld, K., Lewis, D., & McNeill, R. (2020, May 7). Exclusive: Virus exposes gaping holes in Africa’s health systems. *Reuters World News*. Retrieved May 17, 2020, from https://www.reuters.com/article/us-health-coronavirus-africa-response-ex/exclusive-virus-exposes-gaping-holes-in-africas-health-systems-idUSKBN22J1GZ

[CIT0029] Jakhar, D., & Kaur, I. (2020). Potential of chloroquine and hydroxychloroquine to treat COVID-19 causes fears of shortages among people with systemic lupus erythematosus. *Nature Medicine*, *188*, 2020. https://doi.org/10.1038/s41591-020-0853-0 32269358

[CIT0030] Kirby, T. (2020). Evidence mounts on the disproportionate effect of COVID-19 on ethnic minorities. *The Lancet Respiratory Medicine*, *8*(6), 547–548. https://doi.org/10.1016/S2213-2600(20)30228-9 32401711 PMC7211498

[CIT0031] Kones, R., Rumana, U., & Arain, F. (2019). A general pathway model for improving health disparities: Lessons from community and cultural involvement in improving cervical cancer screening in Vietnamese women. *Journal of Clinical Medicine*, *8*(2), 154. https://doi.org/10.3390/jcm8020154 PMC6406352 30700062

[CIT0032] Krumeich, A., & Meershoek, A. (2014). Health in global context; beyond the social determinants of health? *Global Health Action*, *7*(1), 23506. https://doi.org/10.3402/gha.v7.23506 24560252 PMC6442658

[CIT0033] Labonté, R., Mohindra, K., & Schrecker, T. (2011). The growing impact of globalization for health and public health practice. *Annual Review of Public Health*, *32*(1), 263–283. https://doi.org/10.1146/annurev-publhealth-031210-101225 21219153

[CIT0034] Labonté, R., Schrecker, T., Packer, C., & Runnels, V. (2009). Introduction: Globalization’s challenges to people’s health. In *Globalization and health: pathways, evidence and policy*. Routledge.

[CIT0035] Lloyd, M. (2021, January 5). Rising coronavirus cases, limited staff take South Africa’s hospitals to brink of collapse. *ABC News*. Retrieved February 6, 2021, from website: https://www.abc.net.au/news/2021-01-06/south-africa-hospitals-overwhelmed-amid-covid-19-pandemic/13034562

[CIT0036] Lowy Institute. (2021). Covid performance - Lowy Institute. Retrieved February 6, 2021, from https://interactives.lowyinstitute.org/features/covid-performance/

[CIT0037] MacDonald, N. (2009). H1n1 influenza vaccine: Global access for a global problem. *Canadian Medical Association Journal*, *181*(3–4), 123–123. https://doi.org/10.1503/cmaj.091100 19541727 PMC2717664

[CIT0038] Mainous, A. G., Saxena, S., De Rochars, V. M. B., & MacCeus, D. (2020, August 1). COVID-19 highlights health promotion and chronic disease prevention amid health disparities. *British Journal of General Practice*, *70*(697), 372–373. https://doi.org/10.3399/bjgp20X711785 PMC7376756 32690480

[CIT0039] Massinga Loembé, M., Tshangela, A., Salyer, S. J., Varma, J. K., Ouma, A. E. O., & Nkengasong, J. N. (2020, June 11). COVID-19 in Africa: The spread and response. *Nature Medicine*, *26*(7), 999–1003. https://doi.org/10.1038/s41591-020-0961-x 32528154

[CIT0035a] Maxmen, A. (2020, July 21). Ebola prepared these countries for coronavirus – but now even they are floundering. *Nature*, *583*, 667–668. 10.1038/d41586-020-02173-z 32694873

[CIT0040] McMahon, D. E., Peters, G. A., Ivers, L. C., & Freeman, E. E. (2020, July 1). Global resource shortages during COVID-19: Bad news for low-income countries. *PLoS Neglected Tropical Diseases*, *14*(7), 1–3. https://doi.org/10.1371/journal.pntd.0008412 PMC7337278 32628664

[CIT0041] Moloney, A. (2020, May 15). ‘Perfect storm’: Haiti COVID-19 peak set to collide with hurricanes. *National Post.* Retrieved May 17, 2020, from https://nationalpost.com/pmn/environment-pmn/perfect-storm-haiti-covid-19-peak-set-to-collide-with-hurricanes

[CIT0042] Moore, J. T., Ricaldi, J. N., Rose, C. E., Fuld, J., Parise, M., Kang, G. J., Driscoll, A. K., Norris, T., Wilson, N., Rainisch, G., Valverde, E., Beresovsky, V., Agnew Brune, C., Oussayef, N. L., Rose, D. A., Adams, L. E., Awel, S., Villanueva, J., Meaney-Delman, D., … Westergaard, R. (2020). Disparities in incidence of COVID-19 among underrepresented racial/ethnic groups in counties identified as hotspots during June 5–18, 2020 — 22 states, February–June 2020. *MMWR. Morbidity and Mortality Weekly Report*, *69*(33), 1122–1126. https://doi.org/10.15585/mmwr.mm6933e1 32817602 PMC7439982

[CIT0043] Mullard, A. (2020). How COVID vaccines are being divvied up around the world. *Nature*. https://doi.org/10.1038/d41586-020-03370-6 33257891

[CIT0044] Nkengasong, J. N., & Mankoula, W. (2020, March 14). Looming threat of COVID-19 infection in Africa: Act collectively, and fast. *The Lancet*, *395*(10227), 841–842. https://doi.org/10.1016/S0140-6736(20)30464-5 PMC7124371 32113508

[CIT0045] Pai, M. (2020). COVID-19 coronavirus and tuberculosis: We need a damage control plan. *Forbes*, 1–7. https://www.forbes.com/sites/madhukarpai/2020/03/17/covid-19-and-tuberculosis-we-need-a-damage-control-plan/#ee074e7295ca

[CIT0046] Paquette, D. (2020, June 25). The coronavirus is jeopardizing a ‘very, very finite’ workforce: Africa’s doctors and nurses. *The Washington Post*. Retrieved July 15, 2020, from https://www.washingtonpost.com/world/africa/africa-coronavirus-doctors-nurses-health-care-workers/2020/06/25/ebf19256-b4ac-11ea-9b0f-c797548c1154_story.html

[CIT0047] Rawls, J. (1999). *A theory of justice*. Oxford University Press.

[CIT0048] Roberton, T., Carter, E. D., Chou, V. B., Stegmuller, A. R., Jackson, B. D., Tam, Y., Sawadogo-Lewis, T., & Walker, N. (2020). Early estimates of the indirect effects of the COVID-19 pandemic on maternal and child mortality in low-income and middle-income countries: A modelling study. *The Lancet Global Health*, *8*(7), e901–e908. https://doi.org/10.1016/S2214-109X(20)30229-1 32405459 PMC7217645

[CIT0049] Roussi, A., & Maxmen, A. (2020). African nations missing from coronavirus trials. *Nature*. https://doi.org/10.1038/d41586-020-01010-7 32242116

[CIT0050] Rouzier, V., Liautaud, B., & Deschamps, M. M. (2020, July 2). Facing the monster in Haiti. *New England Journal of Medicine*, *383*(1), E4(1)–E4(2). https://doi.org/10.1056/NEJMc2021362 PMC7316259 32543796

[CIT0051] Schemm, P., & Hassan, J. (2021, January 18). WHO chief warns of ‘catastrophic moral failure’ as rich countries dominate vaccine supplies. *The Washington Post*. Retrieved February 6, 2021, from https://www.washingtonpost.com/world/who-chief-warns-of-catastrophic-moral-failure-as-rich-countries-dominate-vaccine-supplies/2021/01/18/033644a0-5978-11eb-a849-6f9423a75ffd_story.html

[CIT0052] Shah, S. (2020, September 1). In race to secure Covid-19 vaccines, World’s poorest countries lag behind. *The Wall Street Journal*. Retrieved September 13, 2020, from https://www.wsj.com/articles/in-race-to-secure-covid-19-vaccines-worlds-poorest-countries-lag-behind-11598998776

[CIT0053] Society of Critical Care Medicine. (2020, May 12). United States resource availability for COVID-19. *Society of Critical Care Medicine Blog*. Retrieved July 4, 2020, from https://sccm.org/Blog/March-2020/United-States-Resource-Availability-for-COVID-19

[CIT0054] Spinello, R. A. (1992). Ethics, pricing and the pharmaceutical industry. *Journal of Business Ethics*, *11*(8), 617–626. https://doi.org/10.1007/BF00872273

[CIT0055] Sternlicht, A. (2020, May 3). Entire stockpile of coronavirus treatment remdesivir donated to government, says CEO. *Forbes*. Retrieved May 18, 2020, from https://www.forbes.com/sites/alexandrasternlicht/2020/05/03/entire-stockpile-of-coronavirus-treatment-remdesivir-donated-to-government-says-ceo/#36c30fb3725f

[CIT0056] Taylor, L. (2020, September 18). Uruguay is winning against covid-19. This is how. *The BMJ*, 370. https://doi.org/10.1136/bmj.m3575 32948599

[CIT0057] The World Bank. (2020a). Jobs data for Haiti: World Bank. *World Bank Jobs Data*. Retrieved July 15, 2020, from http://datatopics.worldbank.org/jobs/country/haiti

[CIT0058] The World Bank. (2020b). Physicians (per 1,000 people). Retrieved May 17, 2020, from https://data.worldbank.org/indicator/SH.MED.PHYS.ZS?view=map&year=2012

[CIT0059] The World Bank. (2020c, April 30). COVID-19 makes handwashing facilities and promotion more critical than ever. *The World Bank*. Retrieved February 6, 2021, from https://www.worldbank.org/en/news/feature/2020/04/30/covid-19-makes-handwashing-facilities-and-promotion-more-critical-than-ever

[CIT0060] Thienemann, F., Pinto, F., Grobbee, D. E., Boehm, M., Bazargani, N., Ge, J., & Sliwa, K. (2020). World heart federation briefing on prevention: Coronavirus disease 2019 (COVID-19) in low-income countries. *Global Heart*, *15*(1). https://doi.org/10.5334/gh.778 PMC7218761 32489804

[CIT0061] Torbay, R. (2020). COVID-19 laid bare how systemic inequities remain. *Health Affairs*, *39*(9), 1656–1656. https://doi.org/10.1377/hlthaff.2020.01248 32897785

[CIT0062] Tosam, M. J., Chi, P. C., Munung, N. S., Oukem-Boyer, O. O. M., & Tangwa, G. B. (2018). Global health inequalities and the need for solidarity: A view from the global south. *Developing World Bioethics*, *18*(3), 241–249. https://doi.org/10.1111/dewb.12182 29266755

[CIT0063] UNICEF. (2020, April 28). COVID-19: Gavi and UNICEF to secure equipment and diagnostics for lower-income countries. *UNICEF Press*. Retrieved May 18, 2020, from https://www.unicef.org/press-releases/covid-19-gavi-and-unicef-secure-equipment-and-diagnostics-lower-income-countries

[CIT0064] U.S Food and Drug Administration. (2020, June 15). Coronavirus (COVID-19) update: FDA revokes emergency use authorization for chloroquine and hydroxychloroquine. *FDA News*. Retrieved June 17, 2020, from https://www.fda.gov/news-events/press-announcements/coronavirus-covid-19-update-fda-revokes-emergency-use-authorization-chloroquine-and

[CIT0065] U.S. National Library of Medicine. (2021). ClinicalTrials.gov. Retrieved January 31, 2021, from https://clinicaltrials.gov/ct2/home

[CIT0066] Wan, W., & Johnson, C. Y. (2020, June 4). The biggest challenge for a coronavirus vaccine could be getting countries to share. *The Washington Post*. Retrieved June 9, 2020, from https://www.washingtonpost.com/health/2020/06/04/biggest-challenge-coronavirus-vaccine-could-be-getting-countries-share/

[CIT0067] Wang, B., Li, R., Lu, Z., & Huang, Y. (2020). Does comorbidity increase the risk of patients with covid-19: Evidence from meta-analysis. *Aging*, *12*(7), 6049–6057. https://doi.org/10.18632/aging.103000 32267833 PMC7185114

[CIT0068] WaterAid. (2020, November 9). WaterAid’s COVID-19 response. Retrieved February 6, 2021, from https://www.wateraid.org/us/wateraids-covid-19-response

[CIT0069] West-Oram, P. G. N., & Buyx, A. (2017). Global health solidarity. *Public Health Ethics*, *10*(2), 212–224. https://doi.org/10.1093/phe/phw021 29731808 PMC5927163

[CIT0070] Whitehead, M. (1992, July 1). The concepts and principles of equity and health. *International Journal of Health Services*, *22*(3), 429–445. https://doi.org/10.2190/986L-LHQ6-2VTE-YRRN 1644507

[CIT0071] Woodyatt, A. (2020, April 18). The world is scrambling to buy ventilators in the Covid-19 pandemic. This country only has four of them. *CNN*. Retrieved May 18, 2020, from https://www.cnn.com/2020/04/18/africa/covid-19-ventilator-shortage-intl-scli/index.html

[CIT0072] World Health Organization. (2018). Addressing the global shortage of, and access to, medicines and vaccines: Report by the Director-General. http://www.unsgaccessmeds.org/final-report

[CIT0073] World Health Organization. (2019, September 13). Hypertension. *World Health Organization*. Retrieved May 17, 2020, from https://www.who.int/news-room/fact-sheets/detail/hypertension

[CIT0074] World Health Organization. (2020a). “Solidarity” clinical trial for COVID-19 treatments. *Global Research on Coronavirus Disease (COVID-19)*. Retrieved July 4, 2020, from https://www.who.int/emergencies/diseases/novel-coronavirus-2019/global-research-on-novel-coronavirus-2019-ncov/solidarity-clinical-trial-for-covid-19-treatments

[CIT0075] World Health Organization. (2020b, April 23). WHO urges countries to move quickly to save lives from malaria in sub-Saharan Africa. *World Health Organization Newsroom*. Retrieved May 17, 2020, from https://www.who.int/news-room/detail/23-04-2020-who-urges-countries-to-move-quickly-to-save-lives-from-malaria-in-sub-saharan-africa

[CIT0076] World Health Organization. (2020c, May 15). Diabetes. *WHO Fact Sheets*. Retrieved May 17, 2020, from https://www.who.int/news-room/fact-sheets/detail/diabetes

[CIT0077] World Health Organization. (2020d, August 24). 172 countries and multiple candidate vaccines engaged in COVID-19 vaccine Global Access Facility. *World Health Organization Newsroom*. Retrieved September 13, 2020, from https://www.who.int/news-room/detail/24-08-2020-172-countries-and-multiple-candidate-vaccines-engaged-in-covid-19-vaccine-global-access-facility

[CIT0078] Worldometer. (2020). Haiti coronavirus. *Worldometer*. Retrieved September 12, 2020, from https://www.worldometers.info/coronavirus/country/haiti/

[CIT0079] Worldometer. (2021). Worldometer coronavirus pandemic. *Worldometers.Info*. Retrieved February 4, 2021, from https://www.worldometers.info/coronavirus/

[CIT0080] Yang, J., Zheng, Y., Gou, X., Pu, K., Chen, Z., Guo, Q., Ji, R., Wang, H., Wang, Y., & Zhou, Y. (2020). Prevalence of comorbidities and its effects in patients infected with SARS-CoV-2: A systematic review and meta-analysis. *International Journal of Infectious Diseases*, *94*, 91–95. https://doi.org/10.1016/j.ijid.2020.03.017 32173574 PMC7194638

[CIT0081] Zimmer, C., Weiland, N., & LaFraniere, S. (2021, February 2). Covid-19: vaccines must be ready to adapt to variants, Fauci says. *The New York Times*. Retrieved February 6, 2021, from https://www.nytimes.com/live/2021/01/29/world/covid-19-coronavirus#johnson-johnsons-vaccine-offers-strong-protection-but-fuels-concern-about-variants

